# Medial Patellofemoral Ligament Repair Augmented With Reinforced Bioinductive Implant Using Double-Bundle Patella Docking Technique

**DOI:** 10.1016/j.eats.2024.103228

**Published:** 2024-09-16

**Authors:** Matthew J. Schultz, Elizabeth Ford, Merrick Wetzler, Sean McMillan

**Affiliations:** a Jefferson Health–New Jersey, Stratford, New Jersey, U.S.A.; b Inspira Health Network, Vineland, New Jersey, U.S.A.; c Advocare South Jersey Orthopedic Associates, Voorhees, New Jersey, U.S.A.; d Virtua Orthopedics, Burlington, New Jersey, U.S.A.

## Abstract

Medial patellofemoral ligament (MPFL) reconstruction with allograft is the current standard surgical treatment for recurrent lateral patellar instability. The authors describe a technique for MPFL repair that is augmented with a reinforced bioinductive implant inserted using a double-bundle patella docking technique. The tensile strength of the implant provides resistance to patellar translation during healing, offering a notable advantage to historically described MPFL repair techniques and paralleling the benefits of allograft over autograft when used for MPFL reconstruction.

In patients with recurrent lateral patellar dislocation, medial patellofemoral ligament repair (MPFLr) or medial patellofemoral ligament reconstruction (MPFLR) is commonly recommended.^1^ Over time, primary MPFLr has fallen out of favor due to high recurrent instability rates.^2^ While MPFLR has demonstrated better outcomes, overall complication rates approach 26%. These complications are multifactorial in terms of surgical technique and graft properties and can lead to acceleration of patellofemoral cartilage damage, loss of motion, and recurrent instability.^3^^,^^4^

Graft choice for MPFLR remains a topic of interest. While semitendinosus allograft is commonly utilized, numerous options have been described.^5^ Graft properties, such as strength and stiffness, are important considerations, particularly given the established risk of overtensioning and increased patellofemoral contact pressure after MPFLR.^3^^,^^4^ Nevertheless, some authors advocate for a graft that is stronger than the native medial patellofemoral ligament (MPFL) to minimize the risk of recurrent instability.^1^

The BioBrace (ConMed) is a reinforced bioinductive implant (RBI) comprising highly porous insoluble bovine type I collagen and bioresorbable poly-L-lactic-acid microfilament yarn. It is indicated for reinforcement of soft tissues where weakness exists. The implant confers 140 N of tensile strength (single stranded, 280 N double stranded) during tissue induction and cellular infiltration phases of healing. It demonstrates linear degradation over a period of 2 years postimplantation. We describe a technique for MPFLr augmented with RBI using a double-bundle patella docking technique.

## Surgical Technique

A video presenting this technique is provided (Video 1).

### Patient Positioning

The patient is positioned supine on a standard operating room table with a nonsterile tourniquet applied at the proximal thigh. The affected leg is prepped and draped in a sterile manner, ensuring exposure to the mid-thigh. A bump can be placed under the ipsilateral buttock. The patient is positioned toward the distal end of the table to allow for fluoroscopic access to the knee, which will be brought in from the contralateral side during use.

### Technique

A standard diagnostic arthroscopy is performed to assess patella maltracking, cartilage damage, and other concurrent pathology. After completion, the tourniquet is inflated to 250 mm Hg, and the knee is flexed to 30° over a sterile triangle. A 3- to 4-cm incision is made over the superomedial aspect of the patella. Careful dissection is performed to the level of the patellar retinaculum. The retinaculum is incised in line with the skin incision at the mid-portion (from superior to inferior) of the patella, preserving a soft tissue sleeve at its patellar insertion for later repair. The superomedial aspect of the patella is exposed, and a Rongeur is used to roughen the medial patella.

Next, 2 pins are placed in parallel fashion at the superior two-thirds of the patella, spaced apart approximately 15 mm. A 4.5-mm cannulated reamer is then placed over the pins and used to over-ream each socket to a depth of approximately 25 mm.

**Fig 1:**
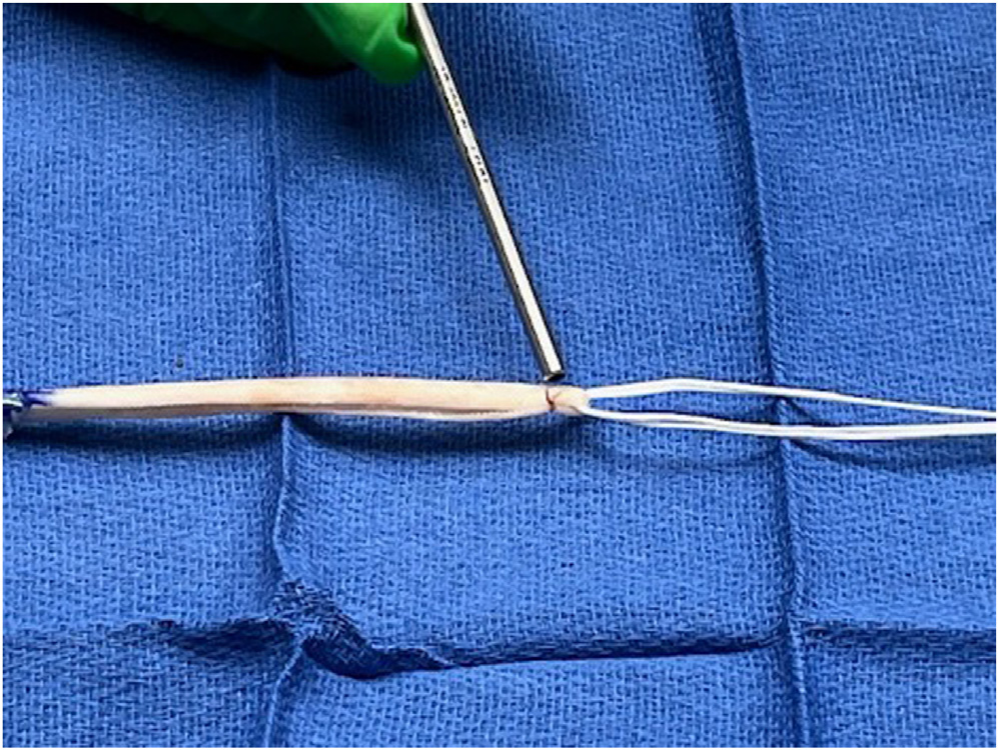
Viewing superiorly, the reinforced bioinductive implant is doubled over and the loop construct is placed at the apex. The reinforced bioinductive implant is doubled over and the loop construct is placed at the apex. A Vicryl suture (metal pointer) is used to tie a simple knot at the apex to secure the loop construct in place and prevent migration from the apex.

After creating the sockets, the RBI is prepared. The implant dimensions are 250 mm long by 5 mm wide. In smaller patients, the authors trim the implant to 220 mm. Each end of the implant is sutured using a propriety graft suture preparation system that can whipstitch the implant in a “Roman Sandle” configuration using a No. 2 nonabsorbable braided ultra-high molecular weight polyethylene suture (SpeedTrap; Depuy Synthes). Next, an adjustable looped-button construct (Infinity Loop; ConMed) is placed in the center of the RBI and the free ends are looped over. A 2-0 Vicryl suture is placed at the apex of the RBI to prevent migration of the looped button construct (Fig 1).

**Fig 2:**
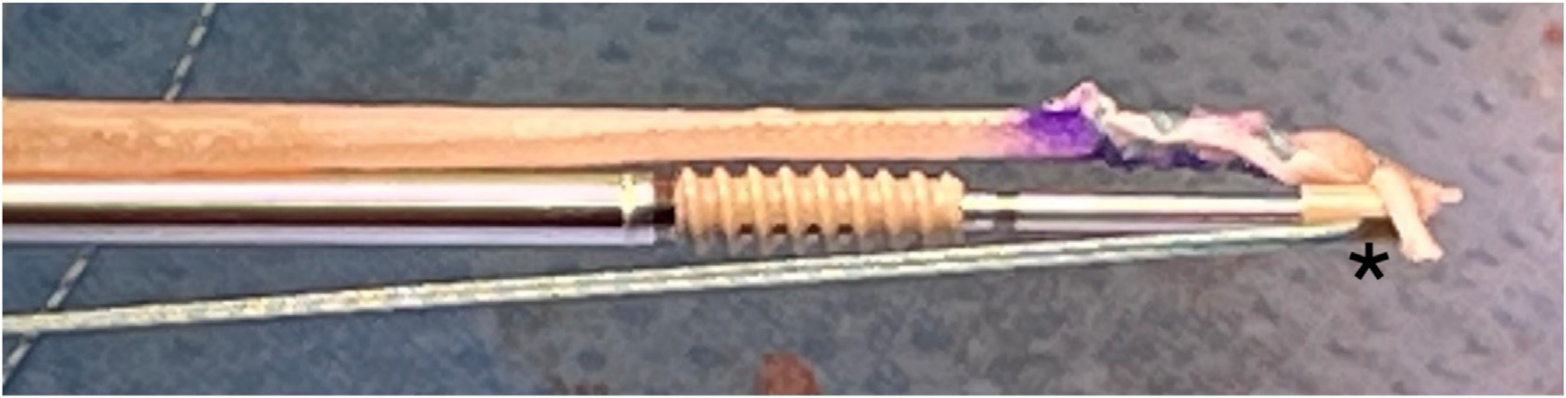
The sutures of the whipstitched end of the reinforced bioinductive implant are placed through the islet of the anchor (∗). The reinforced bioinductive implant is secured tightly up against the polyether ether ketone bone islet tip to ensure complete seating of the implant when placed into the patella sockets.

**Fig 3:**
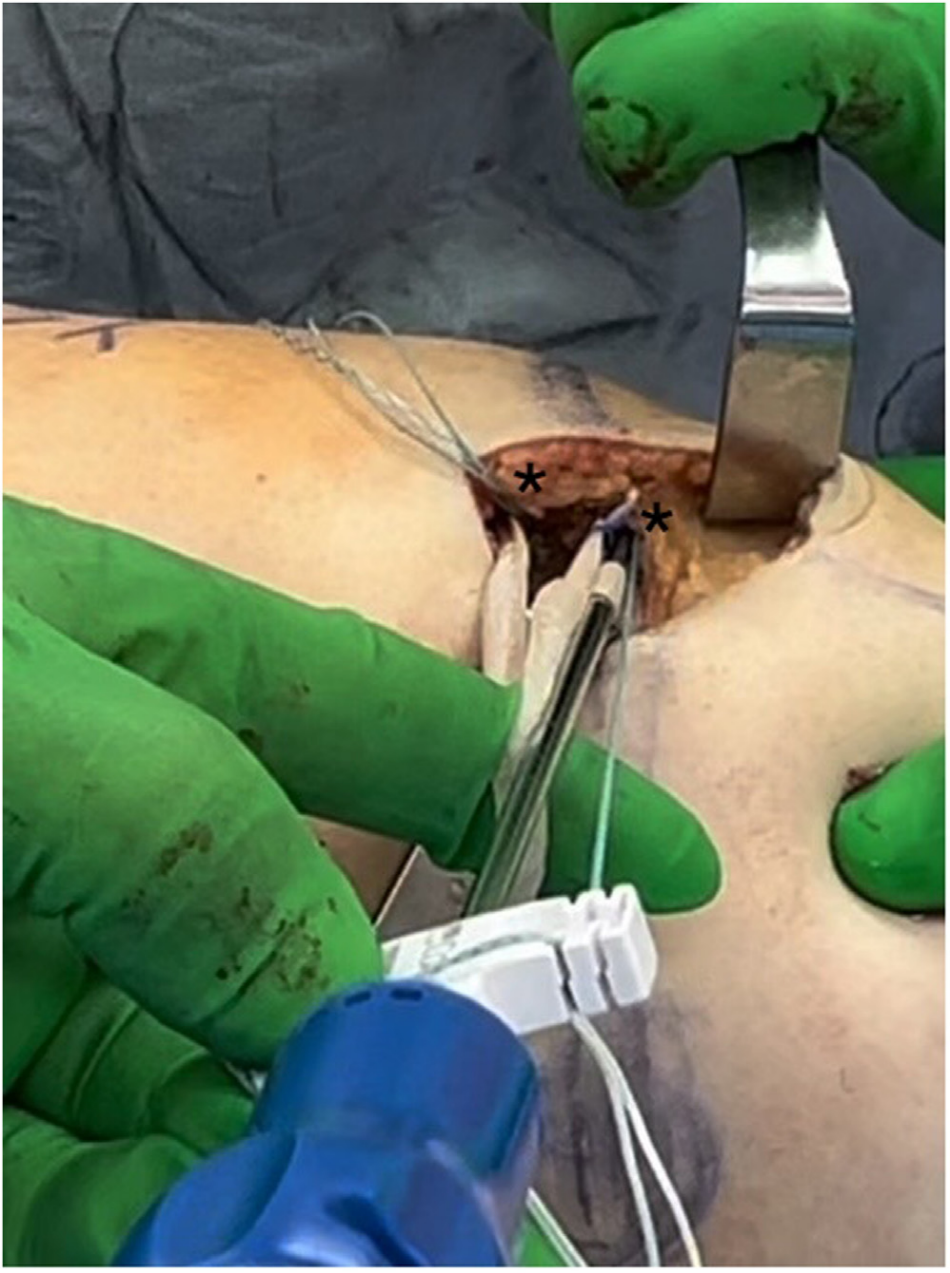
Pictured is a patient in the supine position demonstrating a left knee. The patient's head is out of view to the left of the image and the foot is to the right of the image. Both free ends of the whipstitched reinforced bioinductive implant (RBI) are secured into the patella sockets (∗) using a 4.75-mm polyether ether ketone bone anchor through an incision medial to the patella. Visible is the looped apex of the RBI where the surgeon has placed his index finger. This portion of the RBI will later be passed through layers 2 and 3 on the medial side of the knee and docked into the femoral socket.

After preparation, each free end of the RBI is placed up against a 4.75-mm polyether ether ketone bone anchor (Argo knotless suture anchor; ConMed) with care taken to ensure that the tip of the implant is in line with the tip of the anchor (Fig 2) to allow complete seating of the implant into the socket. The anchor is seated into the predrilled patellar socket with its associated end of the implant (Fig 3). This process is repeated, seating the opposite end of the implant into the second predrilled socket.

**Fig 4:**
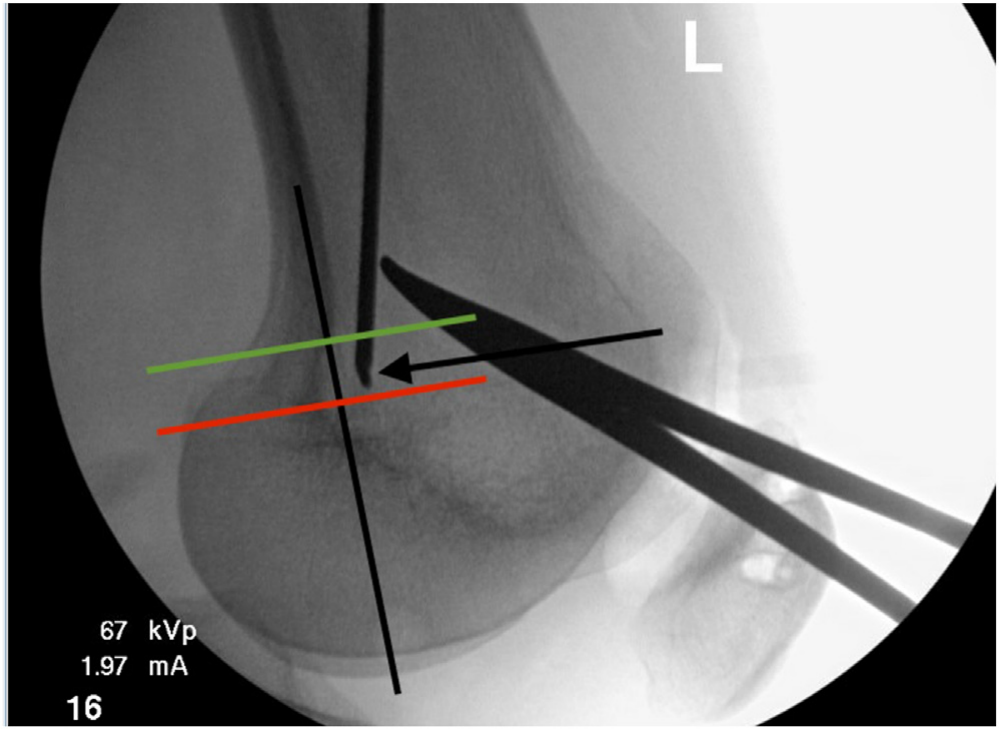
The rendering is of a left knee with the patient in the supine position. The patient's head would be to the right side of the image out of view and the foot would be to the left side of the image. Intraoperative identification of Schottle’s point (arrow). A perfect lateral x-ray is obtained. Schottle’s point is identified just anterior to a line tangent to the posterior femoral cortex (black line) and between horizontal lines between the posterior aspect of Blumensaat’s line (red line) and the superior aspect of the femoral condyle (green line).

The femoral insertion of the MPFL is then identified. A perfect lateral x-ray of the knee is obtained to identify Schottle’s point (Fig 4), and a small medial incision is made at this level. A pin is placed across the femur, starting medially at Schottle’s point and continuing laterally at a trajectory parallel to the articular surface of the knee and the floor until it exits the skin over the lateral thigh. A 6-mm cannulated reamer is placed over the pin and used to over-ream the femoral tunnel unicortically. The pin is removed by pulling it through the lateral side with a shuttling suture loaded through the eyelet to allow for later graft passage.

Next, a shuttling suture is placed between the second and third layers of the medial knee from the medial incision over Schottle’s point to the patella. This is used to pass the looped construct located at the midportion of the implant from the patella to the medial incision. The tails from the looped construct are then passed across the femur with the shuttling suture previously placed through the femoral tunnel. These tails are carefully pulled until the button from the looped construct exits the lateral femoral cortex, and the button is flipped so that it sits flat on the lateral femoral cortex. Button position is confirmed with fluoroscopy.

**Fig 5:**
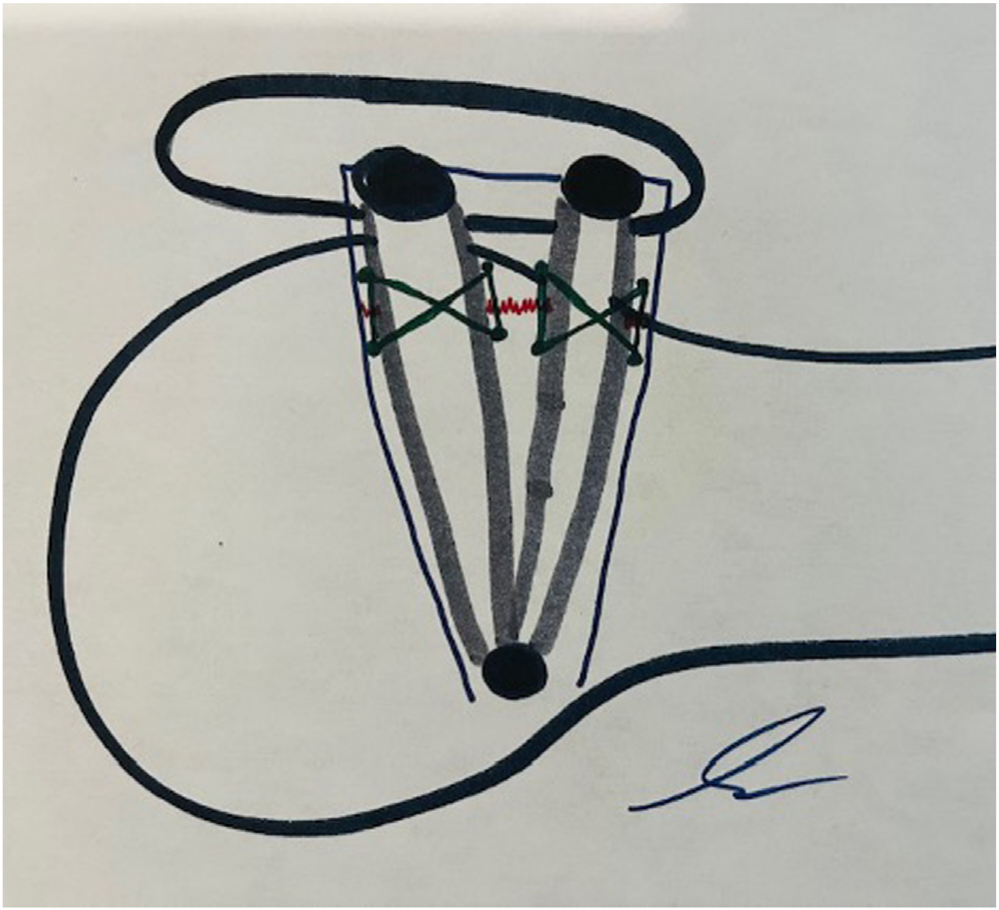
Two No. 2 nonabsorbable suture (green lines) are used to perform the primary repair of the native medial patellofemoral ligament (blue lines) at the site of tear (red lines). The box suture configuration also incorporates the limbs of the reinforced bioinductive implant (gray outlines) for repair augmentation.

**Fig 6:**
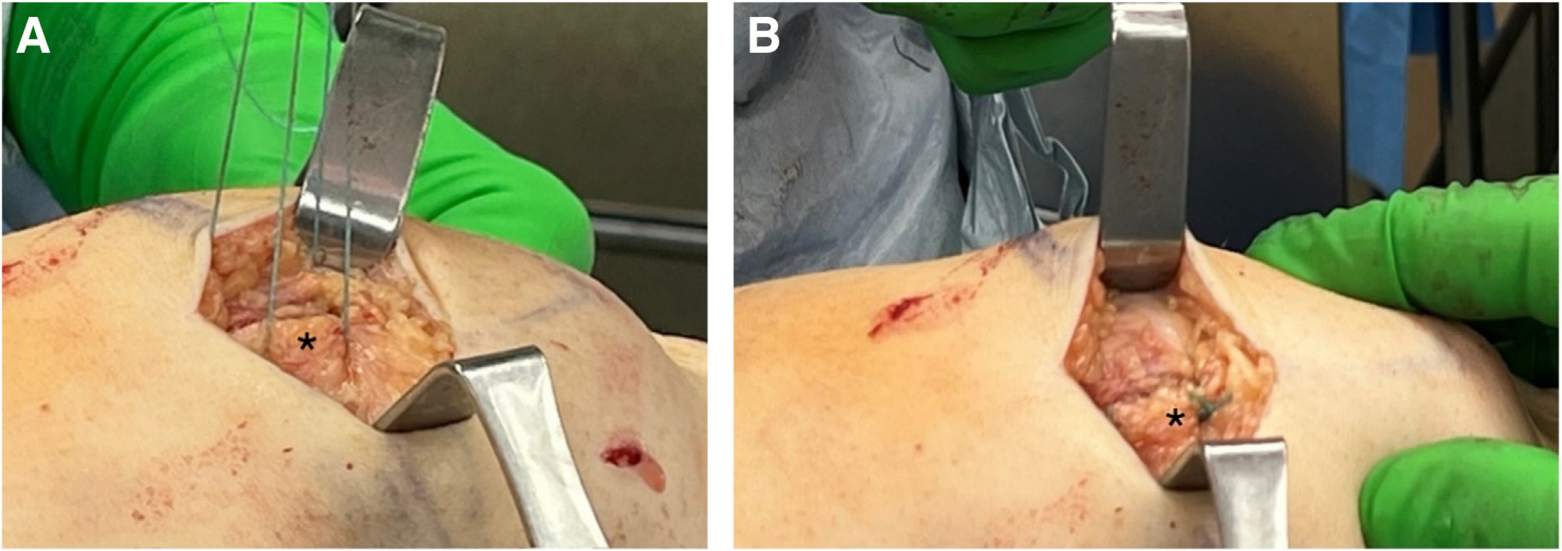
Pictured is a patient in the supine position demonstrating a left knee. The patient's head is out of view to the left of the image and the foot is to the right of the image. Through an incision medial to the patella, the sutures from the ends of the reinforced bioinductive implant are passed back through the patella soft tissue sleeve and the medial retinaculum (asterisk in A), and the final repair is completed in a “pants-over-vest” fashion on the medial side of the knee (asterisk in B).

The knee is then flexed to 30°, and gentle tension is applied through the button on the lateral femoral cortex, taking care not to overtension the construct. If the surgeon feels that overtensioning has occurred, the release button on the adjustable construct can be depressed, and retensioning can be performed (Table 1). Once tensioning is determined, 2 No. 2 nonabsorbable sutures (Dynacord; Depuy Synthes) are placed in a box configuration to repair the torn native MPFL and incorporate the RBI (Fig 5). Next, a free needle is used to pass the suture limbs that were docked into the patella back through the patella soft tissue sleeve and the medial retinaculum in a “pants-over-vest” repair construct (Fig 6 A). The wounds are then irrigated and closed in a standard fashion. The patient is placed into a hinged knee brace with tolerance of 45° of range of motion when at rest for the initial 2 weeks.

**Table 1:** Pearls and Pitfalls of MPFL Repair With RBI Augmentation

Pearls	Pitfalls
Place a Vicryl suture at the apex of the RBI adjacent to the looped construct as demonstrated in Figure 1 to prevent sliding of the loop construct and asymmetric lengths of the 2 bundles of the RBI.	Failure to secure the looped construct at the apex of the RBI prior to passing across the femur. This can result in asymmetric bundle lengths, resulting in 1 tight bundle and 1 loose bundle.
Place the free end of the RBI at the tip of the anchor and securely tension to ensure complete seating of the implant in the socket, as demonstrated in Figure 2.	Incompletely seating the RBI in the patellar sockets. If this occurs, remove the anchor and resecure the RBI at the anchor, as demonstrated in Figure 2.
Tension the RBI with the knee in 30° of flexion.	Overtensioning the RBI. If this occurs, release the tension via the adjustable button “quick-release” and retension.
Confirm that the lateral femoral button is flush against the lateral femoral cortex using fluoroscopy.	Pulling the button through the IT band when passing across the femur to the lateral cortex. If this occurs, make an incision over the lateral exit point of the pin, identify the button sitting over the IT band, longitudinally splint the IT band at this point, and push the button through the IT band to seat it against the lateral femoral cortex.
Ensure femoral position of the RBI is at Schottle’s point using fluoroscopy.	Nonanatomic placement of the RBI. Improper placement can result in persistent instability or stiffness.
Utilize 2 No. 2 sutures to incorporate the RBI into the native MPFL at the location of the tear (femoral or patellar).	
After docking the RBI, use a free needle to pass whipstitched sutures from the patellar end of the RBI into the patella soft tissue and medial retinaculum.	

IT, iliotibial; MPFL, medial patellofemoral ligament repair; RBI, reinforced bioinductive implant.

## Discussion

Currently, the standard surgical treatment for patients with recurrent lateral patellar instability includes anatomic MPFLR.^1^ While the role of femoral and patellar graft placement in the restoration of normal patellofemoral kinematics after these procedures is well understood, graft selection has received relatively less attention.^3^^,^^4^ Some authors advocate for grafts stronger than the native MPFL to compensate for other underlying uncorrected predisposing factors to patellar instability.^1^ However, concern over graft overtensioning and the established risk of patellofemoral arthrosis, which may be secondary to increased contact pressures, raises the question of whether this approach is optimal. With reported complication rates after MPFLR approaching 26%, alternative surgical techniques for lateral patellar instability deserve consideration.^3^, ^4^, ^5^ Table 2 discusses the proposed advantages and disadvantages to MPFLr with RBI augmentation compared to other described techniques.

**Table 2:** Advantages and Disadvantages of MPFL Repair With RBI Augmentation

Advantages	Disadvantages
Lack of donor site morbidity	Need for facility approval to utilize RBI
Predictable graft diameter	Additional cost of RBI compared to autograft
Decreased operative time compared to autograft reconstruction	Uncertainty regarding construct strength compared to traditional allograft MPFL reconstruction technique
Ability of RBI to be stored at room temperature for up to 2 years	
Additional tensile strength conferred by RBI during ligament healing phases compared to standalone repair	

MPFL, medial patellofemoral ligament; RBI, reinforced bioinductive implant.

Historically, MPFLr has demonstrated inferior results to MPFLR in patients with lateral patellar instability. Kruckeberg et al.^2^ reported a 41% redislocation rate in patients treated with MPFLr compared to 14% for MPFLR. Puzzitiello et al.^6^ reported similar redislocation rates, citing a rate of 36.9% for MPFLr compared to 6.3% in MPFLR. Regardless of the reason for instability, the native MPFL fails to provide adequate restraint in a lateral patellar instability population. As such, simple repair of this structure in patients who intend to expose their knee to similar biomechanical stresses going forward seems impractical.

We describe a technique for MPFLr augmented with an RBI using a double-bundle docking technique. The tensile strength of this implant during tissue-healing phases may address the shortcomings of earlier techniques for MPFLr. Biomechanical studies of RBI-augmented infraspinatus repairs compared to nonaugmented controls in an ovine model demonstrated significantly higher load to failure (447 ± 70 N vs 333 ± 51 N, *P* < .01) and lower cyclic creep (1.7 ± 0.2 mm vs 2.2 ± 0.8 mm, *P* = .03) at time zero.^7^ Furthermore, ultimate tensile strength in this model increased between 0, 6, and 12 weeks, ultimately demonstrating strength comparable to contralateral healthy controls at 12 weeks.^8^

Within this Technical Note, we describe a method of MPFLr augmented with an RBI. We feel the use of this implant, which provides tensile strength during tissue-healing phases, confers notable advantages to historically described MPFLr techniques and parallels many of the established benefits of allograft over autograft when used for MPFLR. Future biomechanical and clinical studies should compare the strength and redislocation rates of this construct to primary repair and allograft reconstruction models.

## Disclosures

The authors declare the following financial interests/personal relationships which may be considered as potential competing interests: S.M. reports a relationship with CONMED Corporation that includes consulting or advisory and speaking and lecture fees. All other authors (M.J.S., E.F., M.W.) declare that they have no known competing financial interests or personal relationships that could have appeared to influence the work reported in this paper.

## Supplementary Data

Video 1 Technique for medial patellofemoral ligament repair augmented with reinforced bioinductive implant.
